# Supplementary material for: The interactome of intact mitochondria by cross-linking mass spectrometry provides evidence for coexisting respiratory supercomplexes
Source: Mol Cell Proteomics. 2017 Dec 8;17(2):216–32. doi: 10.1074/mcp.RA117.000470 (PMC5795388; doi:10.1074/mcp.RA117.000470)
Supplement: Supplemental Data [file supp_RA117.000470_133922_0_supp_23978_fzffwf.zip › spectra_annotation/mito_spectra_annotation/129-1-6-1-6-1.pdf]

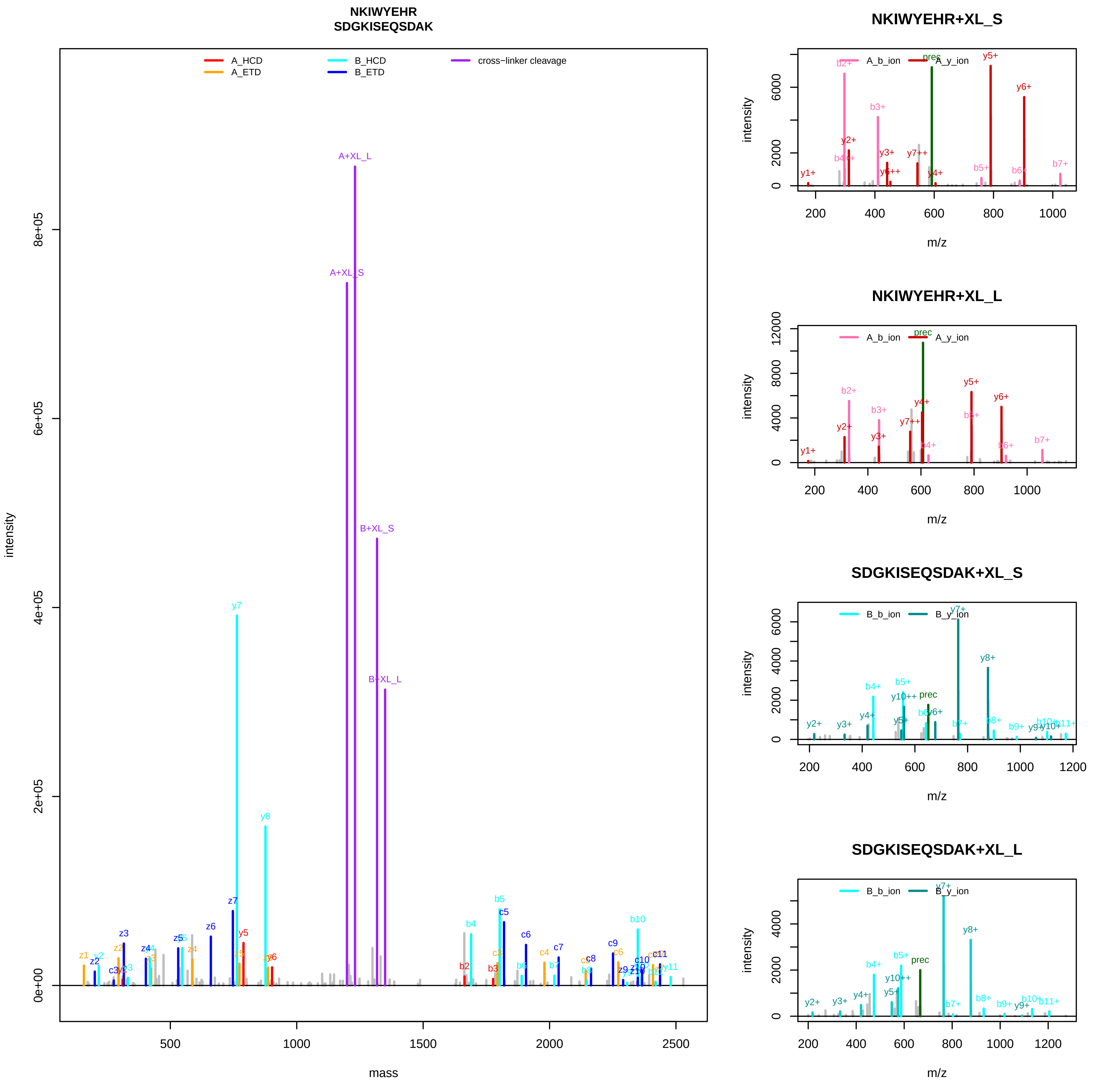
NKIWYEHR
SDGKISEQSDAK
A_HCD
A_ETD
B_HCD
B_ETD
cross−linker cleavage
A+XL_L
A+XL_S
B+XL_S
B+XL_L
y7
y8
z7
y5
z6
z3
z5
b4
b5
c5
b10
c6
c9
c7
c4
c8
c11
intensity
mass
0e+00
2e+05
4e+05
6e+05
8e+05
500
1000
1500
2000
2500
NKIWYEHR+XL_S
A_b_ion
A_y_ion
prec
y5+
y6+
b3+
y2+
y3+
y7++
b5+
b7+
y1+
m/z
NKIWYEHR+XL_L
SDGKISEQSDAK+XL_S
B_b_ion
B_y_ion
y7+
y8+
b4+
b5+
y10++
SDGKISEQSDAK+XL_L
